# High-Dose Hydroxocobalamin for Refractory Vasoplegia Post Cardiac Surgery

**DOI:** 10.7759/cureus.28267

**Published:** 2022-08-22

**Authors:** Marek A Bak, Julian A Smith, Brendan Murfin, Yi Chen

**Affiliations:** 1 Department of Cardiothoracic Surgery, Monash Health, Melbourne, AUS; 2 Intensive Care Unit, Monash Health, Melbourne, AUS

**Keywords:** critical care, vitamin b12, hydroxocobalamin, cardiopulmonary bypass, vasoplegia

## Abstract

Administration of high-dose hydroxocobalamin, or vitamin B12, is an emerging, targeted rescue therapy for the treatment of refractory vasoplegic shock. This is an uncommon but potentially life-threatening complication following cardiac surgery and carries a poor prognosis, particularly when patients fail to respond to first-line therapy with catecholamine vasopressors. This study describes our experience in treating refractory vasodilatory shock following cardiac surgery with high-dose hydroxocobalamin. Administration of hydroxocobalamin in seven patients was associated with an improvement in mean arterial blood pressure or reduction in vasopressor requirements, which were both immediate and sustained throughout our observational period. No deaths or adverse effects attributable to hydroxocobalamin administration occurred in our cohort. Our observations show that high-dose hydroxocobalamin is a safe and effective rescue therapy in refractory vasoplegic shock post cardiopulmonary bypass (CPB).

## Introduction

Vasoplegia is not uncommon following cardiac surgery. It affects 5-25% of patients during or following cardiopulmonary bypass (CPB), with an incidence as high as 50% in patients with predisposing risk factors [1-4]. While in most patients, it resolves without sequelae, the outcome in refractory vasoplegic states is poor, with an estimated mortality of 25% in patients not responsive to first-line vasopressors [1]. Predisposing factors include preoperative antihypertensive use (specifically angiotensin-converting enzyme inhibitors and beta-blockers), low preoperative left ventricular ejection fraction, recent myocardial infarction, and diabetes mellitus [4-6]. No single strict definition exists, but many studies use parameters of low mean arterial pressure (MAP) <65 mmHg, low systemic vascular resistance (SVR) <800 dyne/sec/cm5, and normal-to-high cardiac index (CI) >2.2L/kg/m2 [1,3].

Catecholamine vasopressors are the most common first-line agents used to manage vasoplegia. Other vasoactive drugs used include arginine vasopressin (vasopressin receptor agonist), phenylephrine (alpha-1 receptor agonist), and angiotensin II (angiotensin II receptor type 1 agonist, causing vasoconstriction and upregulating endogenous vasopressin secretion) [7-8]. A 2007 systematic literature review did not demonstrate a clear benefit in the use of any single agent. However, combination therapy using multiple agents with differing mechanisms of action (for example, adrenaline and vasopressin) was shown to be beneficial when a single agent failed to maintain the target blood pressure [7].

Targeted therapies such as methylene blue and hydroxocobalamin are thought to interrupt the cascade of events leading to vasoplegia through their activity on nitric oxide (NO) pathways, which play a key role in the systemic vasodilation seen in vasoplegic and septic shock [1]. Therefore, we aimed to assess the use of high-dose hydroxocobalamin within our unit as rescue therapy for post-cardiotomy refractory vasoplegic shock and to compare this experience with the existing international data.

## Materials and methods

Following review and approval by our Institutional Human Research Ethics Committee, pharmacy dispensing records were used to identify patients who had been administered high-dose hydroxocobalamin in our hospital's ICU following cardiac surgery from January 1, 2019 to October 1, 2021. A total of seven patients were identified, all of whom received it following cardiac surgery. No specific institutional policy exists for selecting patients to receive hydroxocobalamin for treatment of vasoplegia. In all cases, its use was a consensus decision between the treating cardiac surgeon and intensivist for patients requiring high or increasing doses of two or more vasoactive drugs to maintain adequate MAP (aiming over 65 mmHg).

The available form of hydroxocobalamin was Cyanokit (Meridian Medical Technologies, Columbia, MD, USA), containing 5 g of powdered hydroxocobalamin for IV administration. All patients were administered the same dose of 5 g IV hydroxocobalamin, diluted in 200 mL of 0.9% sodium chloride solution, infused over 15 minutes. Administration of hydroxocobalamin took place within the first 24 hours post-operatively in all patients.

Our primary measures of the effects of hydroxocobalamin were MAP and infusion rates of other vasoactive agents. These values were recorded in each patient's electronic medical record by the intensive care nursing staff as part of routine post-cardiac surgery monitoring. However, no specific protocol was in place for additional monitoring or data recording following the administration of hydroxocobalamin.

MAP values and vasopressor infusion rates were recorded from available data in patient charts at the following times: latest pre-administration (mean 31 minutes prior, range 1-52 minutes), earliest post administration (mean 20.5 minutes post, range 2-46 min), at 1-hour post-administration, and 2 hours post-administration. In addition, due to variability in the combinations of vasoactive drugs used, noradrenaline-equivalents (NAE) were calculated for each patient to estimate total vasopressor use in mcg/kg/min. This calculation was based on a previous review paper which determined 0.1 mcg/kg/min of noradrenaline to be equivalent in vasopressor activity to 0.1 mcg/kg/min of adrenaline and 0.04 units/min of vasopressin [9]. 

Patients were followed up for the duration of their index admission: available documentation from the treating surgical and intensive care teams was reviewed for any significant complications attributable to hydroxocobalamin. Subsequently, outpatient follow-up records were reviewed for any potential delayed complications or 30-day mortality.

## Results

Baseline patient characteristics and operative details are presented in Table 1. Four of the seven patients were male, and the mean age was 55 years. One case was elective, while the remainder were urgent inpatient cases. Most patients had two or more pre- or intra-operative risk factors for vasoplegic shock. In the 24 hours prior to surgery, three patients received beta-blockers, and two received ACE-inhibitors. Three had a preoperative LVEF ≤30%. Four patients had a EuroSCORE II greater than 10% (mean 11.7%, range 0.8%-29.6%). Five patients required vasopressor support prior to or while on CPB. The mean duration of CPB was 205 minutes (range 98-528 minutes), and the mean aortic cross-clamp time of 134 minutes (range 51-360 min). No patient required post-operative extracorporeal membrane oxygenation (ECMO) support.

**Table 1:** Baseline characteristics and operative details. F: Female; M: Male; IABP: Intra-aortic balloon pump; CPB: Cardiopulmonary bypass; MAP: Mean arterial pressure; LVEF: Left ventricular ejection fraction; ACE-I: Angiotensin-converting enzyme inhibitor; STEMI: ST-elevation myocardial infarction; NSTEMI: Non-ST-elevation myocardial infarction; AVR: Aortic valve replacement; MVR: Mitral valve replacement; CABG: Coronary artery bypass graft.

Patient	Age (Years)	Sex	Weight (Kg)	BMI	Indication	Surgery	Urgency	IABP inserted intra-op	CPB ime (min)	Aortic cross-clamp time (min)	Redo surgery	Induction MAP (mmHg)	Vasopressors Pre/on CPB	LVEF ≤30% pre-bypass	Dialysis	Hypertension	Diabetes	Beta-blocker pre-op	ACE-I pre-op	Euroscore II mortality estimate (%)
1	58	F	106	42.5	Rheumatic mixed aortic and mitral valve disease	Mechanical AVR + mechanical MVR	Urgent inpatient	N	226	171	N	118	Y	N	N	Y	N	Y	Y	2.4
2	37	F	67	27.9	STEMI, cardiogenic shock	CABG x 2	Emergency	Y	136	55	N	65	Y	Y	N	N	N	N	N	14.2
3	58	M	76	26.0	STEMI	CABG x 3	Emergency	Y	120	51	N	83	Y	Y	N	N	N	N	N	10.3
4	61	M	113	36.5	NSTEMI, ascending aortic aneurysm	CABG x 4 + replacement of ascending aorta	Urgent inpatient	N	163	125	N	90	Y	N	N	Y	N	N	N	6.0
5	48	M	95	28.7	Type A aortic dissection	Replacement of ascending aorta and arch with debranching of innominate and left common carotid arteries + mechanical AVR	Emergency	N	528	360	N	140	Y	N	N	Y	N	Y	Y	29.6
6	56	F	52	20.8	STEMI	CABG x 4	Emergency	N	164	93	N	83	N	Y	N	Y	N	Y	N	19.0
7	67	M	92	30.0	Severe aortic stenosis	Bioprosthetic AVR	Elective	N	98	80	N	110	N	N	N	Y	Y	N	N	0.8

Prior to administration of hydroxocobalamin, all patients were on noradrenaline, five were on adrenaline, and six were on vasopressin. Two patients were on infusions of milrinone, and one was receiving dobutamine. All patients required significant vasopressor doses to maintain a MAP ≥ 65mmHg, with one patient failing to reach this target. The mean NAE was 0.428 mcg/kg/min (range 0.273-0.712 mcg/kg/min). Five patients recorded a baseline CI >2.2. Baseline infusion rates of vasoactive and inotropic agents are listed in Table 2.

**Table 2:** Baseline haemodynamics, vasopressor and inotrope infusion rates. * Data not available MAP: Mean arterial pressure; CI: Cardiac index.

Patient	MAP (mmHg)	CI	Noradrenaline (Mcg/kg/min)	Adrenaline (mcg/kg/min)	Vasopressin (units/Hour)	Milrinone (mcg/kg/min)	Dobutamine (mcg/kg/min)	Total Noradrenaline-Equivalents (NAE) (mcg/kg/min)
1	66	2.46	0.236	0	2.4	0	0	0.336
2	63	2.07	0.478	0.134	2.4	0.125	0	0.712
3	80	2.78	0.276	0.132	2.4	0	2.4	0.508
4	69	3.08	0.177	0.018	2.4	0.375	0	0.295
5	70	*	0.105	0.105	1.5	0	0	0.273
6	72	1.62	0.269	0.231	0.0	0	0	0.500
7	67	3.42	0.326	0.000	1.2	0	0	0.376

**Figure 1:**
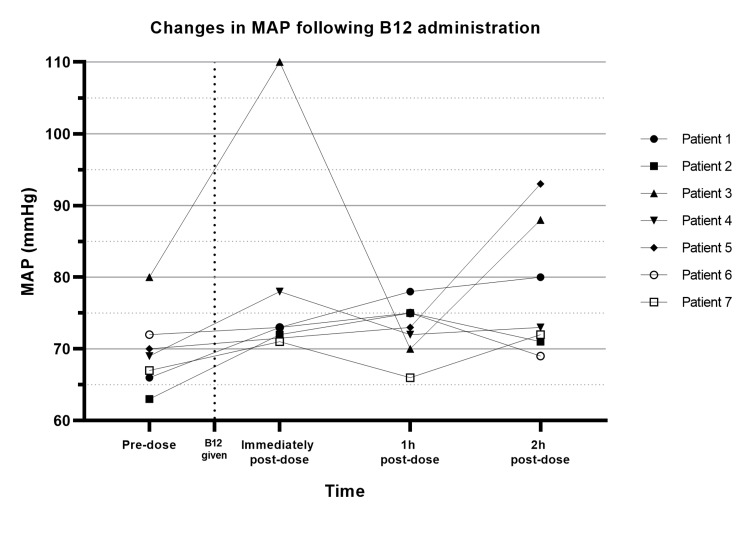
Changes in MAP following B12 administration. MAP: Mean arterial pressure.

**Figure 2:**
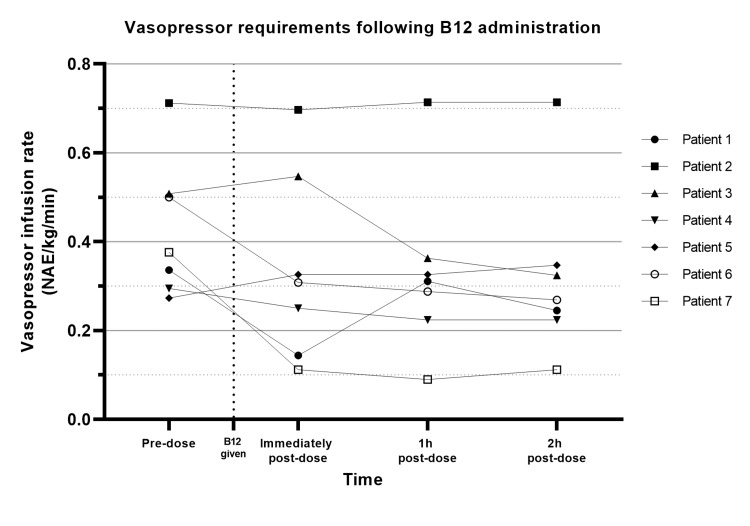
Changes in vasopressor requirements following B12 administration. NAE: Noradrenaline-equivalents.

Patient MAP and total vasopressor infusion rates in the first two hours following hydroxocobalamin administration are outlined in Table 3. These results are represented graphically in Figures 1-2. Notably, the most significant effect on both measurements occurred at the first measurement following administration (recorded at variable times, but all within the first hour). Mean MAP increased by 9.9 mmHg (range 1-30 mmHg), and the mean vasopressor requirement was reduced by 20.5%. At the two-hour mark, a mean change in MAP of +12.1% (range -4.2-32.9%) and vasopressor infusion rates of -25.5% (range -70.2-27%) were recorded. Vasopressor infusion rates were substantially reduced in five patients, unchanged in one patient, and increased in one patient while maintaining a stable or improved MAP. All patients maintained a MAP above 65 mmHg following hydroxocobalamin administration. All patients survived their index admission, and no 30-day mortality was recorded. No adverse effects known to be attributable to this intervention were noted.

**Table 3:** Changes in MAP and vasopressor requirements following B12 administration. * Data not available MAP: Mean arterial pressure; NAE: Noradrenaline-equivalents.

	Pre-dose		First measurement post-dose		1h post dose		2h post dose		Change	
Patient	MAP	Vasopressors	MAP	Vasopressors	MAP	Vasopressors	MAP	Vasopressors	MAP	Vasopressors
1	66	0.336	73	0.144	78	0.311	80	0.245	+ 21.2%	- 27.1%
2	63	0.712	72	0.697	75	0.714	71	0.714	+ 12.70%	+ 0.3%
3	80	0.508	110	0.547	70	0.363	88	0.324	+ 10.0%	- 36.3%
4	69	0.295	78	0.250	72	0.224	73	0.224	+ 5.8%	- 24.0%
5	70	0.273	*	0.326	73	0.326	93	0.347	+ 32.9%	+ 27.0%
6	72	0.500	73	0.308	75	0.288	69	0.269	- 4.2%	- 46.2%
7	67	0.376	71	0.112	66	0.090	72	0.112	+ 7.5%	- 70.2%
Mean	69.6	0.428	79.5	0.341	72.7	0.331	78.0	0.319	+ 12.1%	- 25.5%

## Discussion

Our small case series demonstrated maintenance of adequate MAP (target ≥65mmHg) in all patients, and a significant reduction in vasopressor requirements in 5/7 patients (71%), following the administration of high-dose hydroxocobalamin. One patient had no significant change in their vasopressor requirements, though their MAP increased by 8 mmHg (12.7% increase). These effects were evident shortly after administration and were sustained for at least two hours post-dose.

The pathophysiology of vasoplegic shock is multifactorial, and its underlying processes are often likened to septic shock [10]. In cardiac surgery, direct contact between blood and the CPB circuit induces an inflammatory response mediated by cytokines such as interleukin-1 (IL-1), interleukin-6 (IL-6), and tumor necrosis factor-alpha (TNF-α) [3, 11]. These cytokines activate inducible nitric oxide synthase (iNOS) enzymes, promoting the release of large volumes of NO. This prevents smooth muscle calcium influx and hyperpolarizing smooth muscle cells through activation of adenosine triphosphate (ATP)-sensitive potassium channels, leading to systemic vasodilatation [1, 3]. Prolonged CBP time progressively escalates this response, which is further compounded by CPB-induced hemolysis, protein denaturation, and inflammation from tissue injury due to surgical trauma [3]. In prolonged vasoplegia, the vasoconstrictive effect of intrinsic hormones (adrenaline, noradrenaline, arginine vasopressin, and angiotensin II) is reduced through a combination of hormone depletion, desensitization of adrenergic receptors, and smooth muscle cell hyperpolarisation [12]. This, in turn, may blunt the patient's response to standard vasopressor therapy, typically noradrenaline as a first-line agent [3].

The precise mechanism of hydroxocobalamin's vasoconstrictive effects is unclear, though in vitro studies suggest it acts as a scavenger for NO, binding to it and inhibiting its physiological activity [13]. This is a similar mechanism to methylene blue, another targeted rescue therapy for vasoplegic shock, which acts as a competitive antagonist of NO to inhibit its vasodilatory effects [14]. Hydroxocobalamin also binds to hydrogen sulfide (H2S), an endogenous endothelium-derived hyperpolarizing vasodilator produced through the metabolism of homocysteine [15].

Our findings are consistent with currently published studies. Small-scale retrospective studies have demonstrated that high-dose hydroxocobalamin appears to be a safe and effective treatment for managing CPB-associated vasoplegia. A 2018 systematic review identified that most patients showed an immediate and sustained improvement in MAP and reduction in vasopressor requirements following hydroxocobalamin administration, with no serious adverse events attributed to its use [15]. A further case series published in 2019 reported similar findings, with immediate improvement in blood pressure, followed by a sustained reduction in ongoing vasopressor requirements [16]. Given its off-label use in treating vasoplegia, the dosing of this drug in our center is based on its primary indication of cyanide poisoning - 5-10 g IV infusion over 15 minutes. A recently published case series from the USA investigated the effects of extended duration infusion of the same 5 g dose (median 6h, range 1-10h), finding a significant and gradual reduction of vasopressor requirements throughout the infusion, which appeared to be sustained after cessation [17]. Further studies may be helpful in determining optimal dosing for the indication of vasoplegic shock.

No significant adverse events attributable to hydroxocobalamin were noted in our cohort. According to product information and currently published data, serious complications are rare, and the drug is well tolerated [15]. However, recognized adverse effects include rash, nausea, headaches, infusion site reactions, hypertension, and the formation of oxalate crystals in the urine [18]. Additionally, the deep red colour of the drug may cause chromaturia, interference with some colorimetric laboratory tests, and pigmentation of hemodialysis ultrafiltrate, which may cause an erroneous blood leak detection alarm on some hemodialysis machines [15, 19].

Two recent retrospective cohort studies have been published comparing hydroxocobalamin with methylene blue in refractory vasoplegic shock. A 2020 study of 35 patients identified a significant increase in both MAP and SVR within the first hour after administration of both agents, with only hydroxocobalamin being associated with an ongoing reduction in vasopressor requirements at one- and four-hours post-dose [20]. However, another study of 88 patients showed no significant difference between the effectiveness of hydroxocobalamin and methylene blue in reducing overall vasopressor requirements [21].

The major limitations of this study are the retrospective nature of the analysis, lack of a control group, and a small number of patients enrolled. It is important to note that shock following cardiac surgery may have multiple underlying etiologies (vasodilatory, hypovolaemic, cardiogenic). Clinical judgment is required in patients with borderline cardiac output in whom increasing vascular resistance may unmask or exacerbate cardiogenic shock. Our findings, along with previously published literature, support the use of hydroxocobalamin as safe and effective rescue therapy in post-CPB vasoplegic shock. No randomized trials have been published. The question of whether this improves overall patient outcomes remains unanswered. We would propose a prospective controlled study protocol targeting high-risk patients requiring increasing doses of two or more vasopressors to maintain a MAP target of ≥65mmHg despite adequate fluid resuscitation with normal or increased cardiac output.

## Conclusions

High-dose hydroxocobalamin injection appears to be a safe and effective rescue therapy for patients in vasoplegic shock following CPB. Its use is associated with a significant, immediate, and sustained reduction in vasopressor requirements while maintaining adequate MAP, with no significant adverse effects noted in our study population. Further investigation is warranted with a randomized controlled trial to better assess the effect of hydroxocobalamin on patient outcomes.
